# Supplementary material for: Generation of a Novel SARS-CoV-2 Sub-genomic RNA Due to the R203K/G204R Variant in Nucleocapsid: Homologous Recombination has Potential to Change SARS-CoV-2 at Both Protein and RNA Level
Source: Pathog Immun. 2021 Aug 20;6(2):27–49. doi: 10.20411/pai.v6i2.460 (PMC8439434; doi:10.20411/pai.v6i2.460)
Supplement: Supplemental Figures 1-5 [file pai-6-027-s01.pdf]

Generation of a novel SARS-CoV-2 sub-genomic RNA due to the R203K/G204R variant in nucleocapsid: homologous recombination has potential to change SARS-CoV-2 at both protein and RNA level

Authors

Shay Leary[1¶], Silvana Gaudieri[1,2,3¶], Matthew D. Parker[4¶], Abha Chopra[1], Ian James[1], Suman Pakala[3], Eric Alves[2], Mina John[1,5], Benjamin B. Lindsey[6,7], Alexander J Keeley[6,7], Sarah L. Rowland-Jones[6,7], Maurice S. Swanson[8], David A. Ostrov[9], Jodi L. Bubenik[8], Suman Das[3], John Sidney[10], Alessandro Sette[10,11], COVID-19 Genomics Consortium UK, Thushan I. de Silva[6,7*], Elizabeth Phillips[1,3*], Simon Mallal[1,3#*]

Affiliated Institutions

[1]Institute for Immunology and Infectious Diseases, Murdoch University, Murdoch, Western Australia, Australia.
[2]School of Human Sciences, University of Western Australia, Crawley, Western Australia, Australia.
[3]Division of Infectious Diseases, Department of Medicine, Vanderbilt University Medical Center, Nashville, Tennessee, United States.
[4]Sheffield Biomedical Research Centre, Sheffield Bioinformatics Core, The University of Sheffield, Sheffield, United Kingdom.
[5]Department of Clinical Immunology, Royal Perth Hospital, Perth, Western Australia, Australia.
[6]Sheffield Teaching Hospitals NHS Foundation Trust, Sheffield, United Kingdom.
[7]Department of Infection, Immunity and Cardiovascular Disease and The Florey Institute for Host-Pathogen Interactions, Medical School, University of Sheffield, Sheffield, United Kingdom.
[8]Department of Molecular Genetics and Microbiology, Center for NeuroGenetics and the Genetics Institute, University of Florida, Gainesville, Florida, United States.
[9]Department of Pathology, Immunology and Laboratory Medicine, University of Florida, Gainesville, Florida, United States.
[10]Center for Infectious Disease and Vaccine Research, La Jolla Institute for Immunology, La Jolla, California, United States.
[11]Department of Medicine, Division of Infectious Diseases and Global Public Health, University of California, San Diego, La Jolla, California, United States.

[¶]These authors contributed equally to this work.
[*]These authors also contributed equally to this work.

[#]Corresponding Author
Prof. Simon Mallal
Email: s.mallal@vumc.org

DOI: 10.20411/pai.v6i2.460

This file includes:

Figures S1 to S5

Accession numbers:

Metatranscriptome data from coronaviruses in acute respiratory infections and asymptomatic subjects:

Coronavirus_NL63_S168.sqn	PRJNA671738	SAMN16547776	SRR12893437
Coronavirus_NL63_S170.sqn	PRJNA671738	SAMN16547777	SRR12893436
Coronavirus_OC43_S219.sqn	PRJNA671738	SAMN16547778	SRR12893435
Coronavirus_229E_S220.sqn	PRJNA671738	SAMN16547779	SRR12893434

Data for clinical cohort at https://www.cogconsortium.uk/data/.

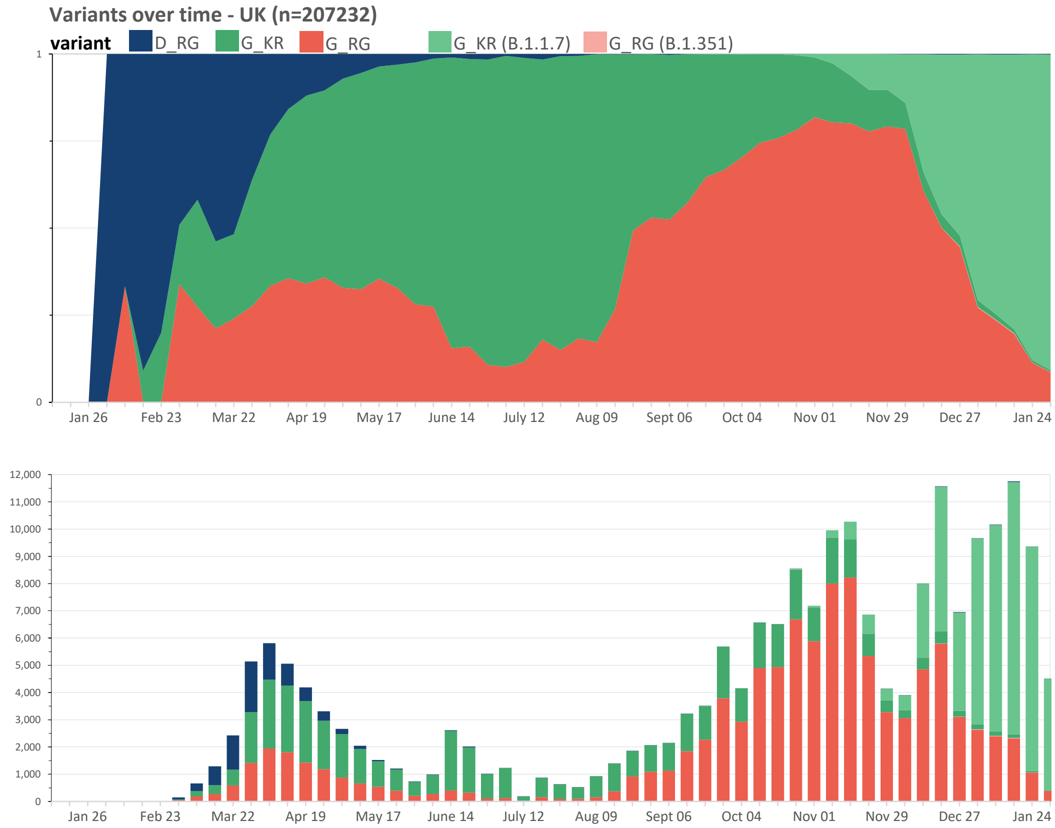

Figure S1. Proportion of weekly deposited SARS-CoV-2 sequences in the UK. Top panel is the proportion of different strains. Bottom panel is the number of deposited sequences in the GISAID database broken down into specific variants. The B.1.1.7 ‘UK variant’ is the main deposited strain in recent months. D_RG = D614/R203/G204; G_RG = G614/R203/G204; G_KR = G614/K203/R204; G_KR (B.1.1.7) = ‘UK variant’; and G_RG (B.1.351) = ‘South African variant’.

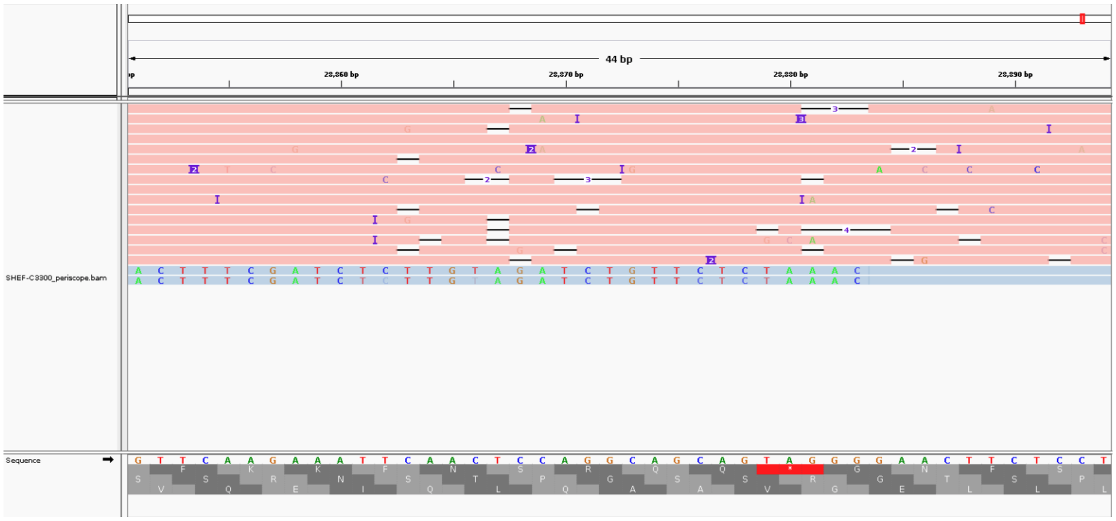

Figure S2. Possible erroneous demultiplexing is responsible for novel sgRNA detected in a single sequence with R203/G204. This sample does not have the R203K/G204R mutation but two reads were classified as novel sgRNA by the periscope tool (blue). Further investigation shows that this sample, indeed, does not contain any evidence of the new TRS (red reads). It is possible that these reads are due to a barcoding issue or a very low level of sample contamination.

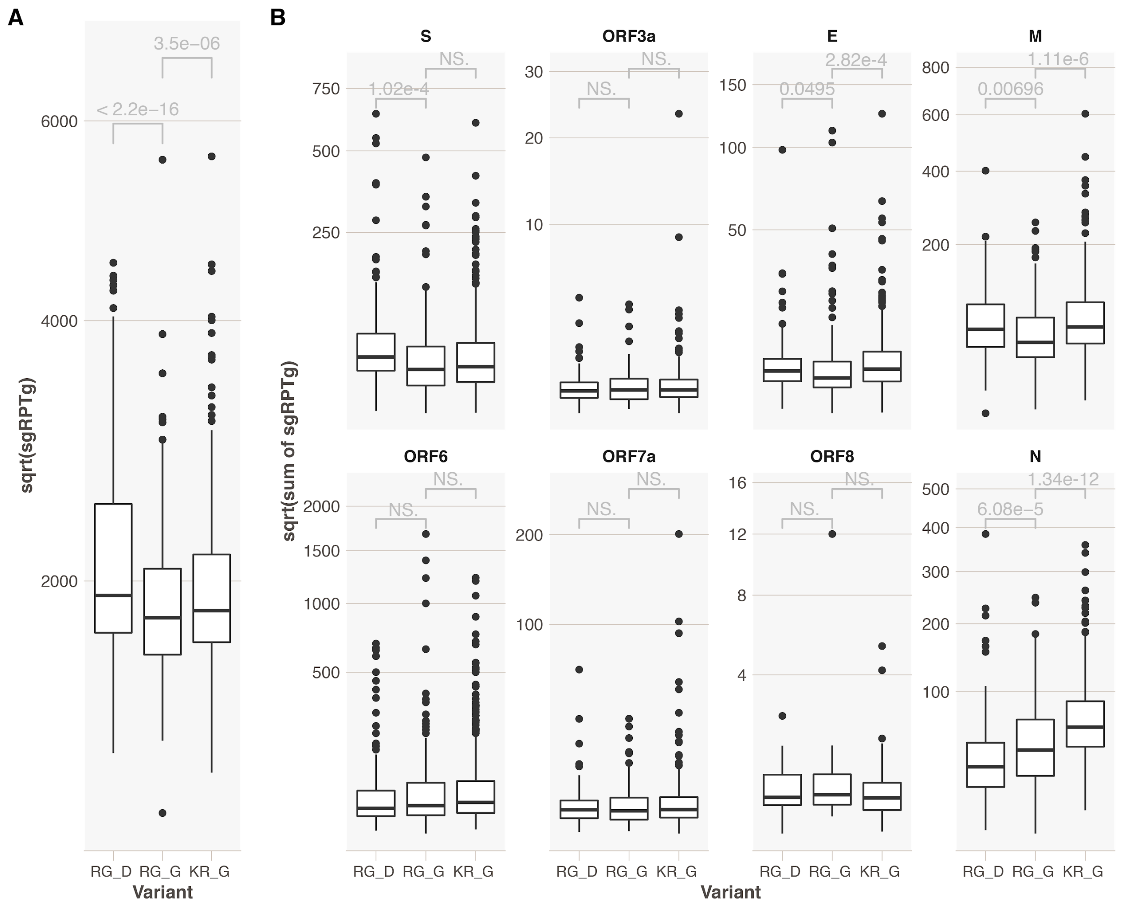

Figure S3. Sub-genomic RNA levels considering the D614 site in spike protein. Samples with a D at residue 614 in the spike protein appear to have increased expression of sgRNA compared to those with a G at this residue. (A). Across all sgRNAs and (B) in selected individual sgRNAs (S = spike, M = membrane and E = envelope). Y-axis is square root transformed sgRNA reads per 1000 genomic RNA reads from corresponding amplicons. p values from Mann-Whitney U, adjusted for multiple testing with the Holm method. RG = R203/G204 containing variant; KR = K203/R204 containing variants; D = spike D614 containing variants, G = spike G614 containing variants.

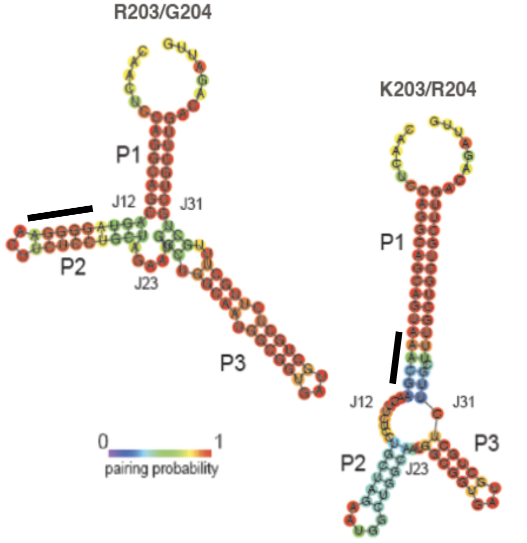

Figure S4. Predicted RNA structures corresponding to the R203/G204 and K203/R204 forms of the nucleocapsid suggest alterations to the three-way junctions. Predictions performed using the RNAfold program with pairing probability shown. Black bars highlight the 203/204 codons.

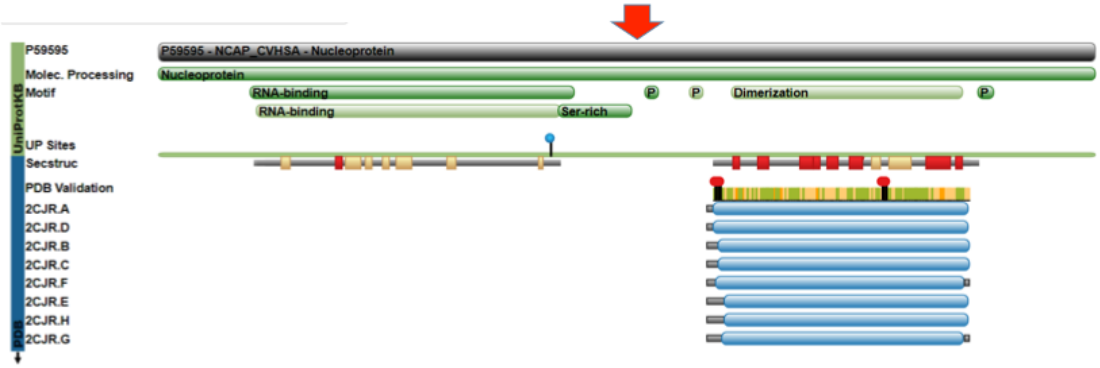

Figure S5 Location of R203K/G204R polymorphisms between the RNA-binding and dimerization domains. The grey horizontal bar indicates the full length nucleocapsid protein with the individual domains and specific regions indicated by green horizontal bars. The left side panel indicates the presence of structures in the public databases. There are no structures for the region adjacent to the serine-rich stretch and containing the R203K/G204R sites (indicated by a red arrow). Structures are available for the RNA-binding domain (including for SARS-CoV-2) and dimerization domain (SARS-CoV only).
