# Supplementary material for: Generation of a Novel SARS-CoV-2 Sub-genomic RNA Due to the R203K/G204R Variant in Nucleocapsid: Homologous Recombination has Potential to Change SARS-CoV-2 at Both Protein and RNA Level
Source: Pathog Immun. 2021 Aug 20;6(2):27–49. doi: 10.20411/pai.v6i2.460 (PMC8439434; doi:10.20411/pai.v6i2.460)
Supplement: Supplemental Tables 1-9 [file pai-6-027-s02.pdf]

Generation of a novel SARS-CoV-2 sub-genomic RNA due to the R203K/G204R variant in nucleocapsid: homologous recombination has potential to change SARS-CoV-2 at both protein and RNA level

Authors

Shay Leary[1¶], Silvana Gaudieri[1,2,3¶], Matthew D. Parker[4¶], Abha Chopra[1], Ian James[1], Suman Pakala[3], Eric Alves[2], Mina John[1,5], Benjamin B. Lindsey[6,7], Alexander J Keeley[6,7], Sarah L. Rowland-Jones[6,7], Maurice S. Swanson[8], David A. Ostrov[9], Jodi L. Bubenik[8], Suman Das[3], John Sidney[10], Alessandro Sette[10,11], COVID-19 Genomics Consortium UK, Thushan I. de Silva[6,7*], Elizabeth Phillips[1,3*], Simon Mallal[1,3#*]

Affiliated Institutions

[1]Institute for Immunology and Infectious Diseases, Murdoch University, Murdoch, Western Australia, Australia.
[2]School of Human Sciences, University of Western Australia, Crawley, Western Australia, Australia.
[3]Division of Infectious Diseases, Department of Medicine, Vanderbilt University Medical Center, Nashville, Tennessee, United States.
[4]Sheffield Biomedical Research Centre, Sheffield Bioinformatics Core, The University of Sheffield, Sheffield, United Kingdom.
[5]Department of Clinical Immunology, Royal Perth Hospital, Perth, Western Australia, Australia.
[6]Sheffield Teaching Hospitals NHS Foundation Trust, Sheffield, United Kingdom.
[7]Department of Infection, Immunity and Cardiovascular Disease and The Florey Institute for Host-Pathogen Interactions, Medical School, University of Sheffield, Sheffield, United Kingdom.
[8]Department of Molecular Genetics and Microbiology, Center for NeuroGenetics and the Genetics Institute, University of Florida, Gainesville, Florida, United States.
[9]Department of Pathology, Immunology and Laboratory Medicine, University of Florida, Gainesville, Florida, United States.
[10]Center for Infectious Disease and Vaccine Research, La Jolla Institute for Immunology, La Jolla, California, United States.
[11]Department of Medicine, Division of Infectious Diseases and Global Public Health, University of California, San Diego, La Jolla, California, United States.

[¶]These authors contributed equally to this work.
[*]These authors also contributed equally to this work.

[#]Corresponding Author

Prof. Simon Mallal
Email: s.mallal@vumc.org

DOI: 10.20411/pai.v6i2.460

This file includes:

Tables S1 to S9

Accession numbers:

Metatranscriptome data from coronaviruses in acute respiratory infections and asymptomatic subjects:

Coronavirus_NL63_S168.sqn	PRJNA671738	SAMN16547776	SRR12893437
Coronavirus_NL63_S170.sqn	PRJNA671738	SAMN16547777	SRR12893436
Coronavirus_OC43_S219.sqn	PRJNA671738	SAMN16547778	SRR12893435
Coronavirus_229E_S220.sqn	PRJNA671738	SAMN16547779	SRR12893434

Data for clinical cohort at https://www.cogconsortium.uk/data/.

Table S1. Amino acid variations and alternative codon usage of SARS-CoV-2 (>5% frequency of deposited sequences; 24th January 2021).

Gene / Protein [Length] Amino Acid Position	Codon Usage	
**ORF1ab / ORF1ab protein [7097]**	**Amino Acid [Codon Count] (5% cutoff)**	
60	V [GTT 78] [GTC 21]	
216	S [TCC 88] [TCT 11]	
265	T [ACC 85]	I [ATC 14]
924	F [TTT 94] [TTC 5]	
1001	T [ACT 88]	I [ATT 11]
1708	A [GCT 88]	D [GAT 11]
1907	F [TTC 87] [TTT 12]	
2007	T [ACC 78] [ACT 21]	
2230	I [ATA 88]	T [ACA 11]
3606	L [TTG 94]	F [TTT 5]
4715	L [CTT 94]	P [CCT 5]
4804	P [CCC 88] [CCT 11]	
5005	H [CAC 88] [CAT 11]	
5304	T [ACT 88] [ACC 11]	
6205	L [CTA 94] [TTA 5]	
6668	L [TTA 93] [TTG 6]	
6997	A [GCG 77] [GCC 22]	

**S / surface protein [1274]**	**Amino Acid [Codon Count] (5% cutoff)**	
18	L [CTT 90]	F [TTT 9]
222	A [GCT 77]	V [GTT 22]
477	S [AGC 94]	N [AAC 5]
501	N [AAT 87]	Y [TAT 12]
570	A [GCT 88]	D [GAT 11]
614	G [GGT 94]	D [GAT 5]
681	P [CCT 87]	H [CAT 12]
716	T [ACA 88]	I [ATA 11]
982	S [TCA 88]	A [GCA 11]
1118	D [GAC 88]	H [CAC 11]

**ORF3a [276]**	**Amino Acid [Codon Count] (5% cutoff)**	
57	Q [CAG 78]	H [CAT 21]

**M / membrane glycoprotein [223]**	**Amino Acid [Codon Count] (5% cutoff)**	
71	Y [TAC 94] [TAT 5]	
93	L [CTC 77] [CTG 21]	

**ORF8 / OFR8 protein [122]**	**Amino Acid [Codon Count] (5% cutoff)**	
17	H [CAC 85] [CAT 15]	
24	S [TCA 94]	L [TTA 5]
27	Q [CAA 88]	* [TAA 11]

**52**	R [AGA 88]	I [ATA 11]
**73**	Y [TAC 88]	C [TGC 11]

N / nucleocapsid [421]	Amino Acid [Codon Count] (5% cutoff)	
**3**	D [GAT 88]	L [CTA 11]
**194**	S [TCA 94]	L [TTA 5]
**199**	P [CCA 94]	L [CTA 5]
**203**	R [AGG 62]	K [AAA 37]
**204**	G [GGA 62]	R [CGA 37]
**220**	A [GCT 78]	V [GTT 21]
**235**	S [TCT 88]	F [TTT 11]

ORF10 [276]	Amino Acid [Codon Count] (5% cutoff)	
**30**	V [GTA 78]	L [TTA 21]

Table S2. Peptide prediction for regions in SARS-CoV-2 containing the R203K/G204R amino acid combinations.*

NetMHC	HLA	Peptide	1-log50k(aff)	Affinity(nM)	%Rank	Bind Level
KR	HLA-A 30:01	**R**TSPARMAG	0.603	73.42	0.5	SB
RG	HLA-A 30:01	**G**TSPARMAG	0.342	1235.69	2.5	
KR	HLA-A 68:01	NSTPGSS**KR**	0.666	37.12	0.5	SB
RG	HLA-A 68:01	NSTPGSS**RG**	0.063	25298.77	22	
KR	HLA-B 15:03	S**KR**TSPARM	0.725	19.68	0.3	SB
RG	HLA-B 15:03	S**RG**TSPARM	0.273	2606.69	6	
KR	HLA-B 73:01	**KR**TSPARMA	0.166	8306.36	0.5	SB
RG	HLA-B 73:01	**RG**TSPARMA	0.035	34081.84	11	
KR	HLA-C 07:01	S**KR**TSPARM	0.104	16237.59	6.5	
RG	HLA-C 07:01	S**RG**TSPARM	0.248	3406.51	1.2	WB
KR	HLA- C07:02	S**KR**TSPARM	0.216	4831.5	1.5	WB
RG	HLA- C07:02	S**RG**TSPARM	0.296	2035.83	0.7	WB

NetMHCpan	HLA	Peptide	Score	%Rank	Bind Level
KR	HLA-A 30:01	RNSTPGSS**K**	0.281867	0.4783	SB
RG	HLA-A 30:01	RNSTPGSS**R**	0.092325	2.6775	
KR	HLA-A 30:01	**R**TSPARMAG	0.438433	0.1607	SB
RG	HLA-A 30:01	**G**TSPARMAG	0.162082	1.3184	WB
KR	HLA-A 68:01	NSTPGSS**KR**	0.705615	0.1942	SB
RG	HLA-A 68:01	NSTPGSS**RG**	0.00035	35.9355	
KR	HLA-A 33:03	NSTPGSS**KR**	0.378816	0.3332	SB
RG	HLA-A 33:03	NSTPGSS**RG**	0.000122	54.0541	
KR	HLA-C 07:01	S**KR**TSPARM	0.041824	3.6073	
RG	HLA-C 07:01	S**RG**TSPARM	0.318753	0.3051	SB
KR	HLA-C 07:02	S**KR**TSPARM	0.059982	4.5638	
RG	HLA-C 07:02	S**RG**TSPARM	0.355166	0.5579	WB
KR	HLA-B 38:01	S**KR**TSPARM	0.003955	13.1727	
RG	HLA-B 38:01	S**RG**TSPARM	0.080137	1.8794	WB
KR	HLA-B 14:02	S**KR**TSPARM	0.043487	3.2691	
RG	HLA-B 14:02	S**RG**TSPARM	0.080815	1.7515	WB

**SB = strong binder and WB = weak binder. Sites 203 and 204 indicated in red.*

Table S3. Binding affinity of peptides to specific HLA alleles including peptides containing the R203K/G204R variants.

					Binding affinity (IC50 nM)	
Peptide ID	Sequence	Len	Source	Notes	B*08:01	C*07:01
1054.0002	FLRGRAYGI	9	HSV nuc 11	HLA B8 T cell epitope	**0.85**	-
4199.0002	QAKWRLQTL	9	HSV nuc 26	B08 tetramer; PMID:11927633	**2.4**	-
4199.0001	EIYKRWII	8	HIV gag 260	B08 tetramer; PMID:27760342	**2.6**	-
960.0002	FLKDYQLL	8	HIV gp 586	Analog of B8 epitope	**6.9**	-
3484.0028	IRSSYIRVL	9	Mamu DNA rep factor 289	Mamu B*1001/HLA C*07:01 ligand	**301**	**0.21**
4196.0001	YQSGLSIVM	9	MTB hyp protein 48	C*0701 binder; PMID:23555576	1107	**67**
4196.0002	ANNTRLWVY	9	MTB ag 85B	C*0701 tetramer; PMID:25809751	42748	1600
1074.0001	YTAVVPLVY	9	Hu J chain 102	HLA A1 eluted ligand	29285	1279
4197.0001	SRGTSPARM	9	SARS-CoV-2 nuc 202		-	-
4197.0002	SKRTSPARM	9	SARS-CoV-2 nuc 202		10620	-

A dash indicates IC50 >50000 nM.

Table S4. Frequency of linked amino acid variations across the SARS-CoV-2 genome (>0.01% frequency of deposited sequences; 24th January 2021).*

N		ORF1ab									S													ORF3	ORF8				N					ORF10	
KR#	RG#	T 265 I	T 1001 I	A 1708 D	I 2230 T	L 3606 F	SGF 3675-3577 deletion			L 4715 P	L 18 F	HV 69-70 deletion		Y 144 deletion	A 222 V	S 477 N	N 501 Y	A 570 D	G 614 D	P 681 H	T 716 I	S 982 A	D 1118 H	Q 57 H	S 24 L	Q 27 *	R 52 I	Y 73 C	D 3 L	S 194 L	P 199 L	A 220 V	S 235 F	V 30 L	
		1058	3266	5387	6953	11081	11288	11291	11294	14407	21614	21767	21770	21992	22226	22991	23063	23270	23402	23603	23708	24506	24914	25561	27963	27972	28047	28110	28280	28853	28868	28931	28976	29645	28880 - 28885
0	11834	T	T	A	I	L	S	G	F	P	L	H	V	Y	A	S	N	A	D	P	T	S	D	Q	S	Q	R	Y	D	S	P	A	S	V	WILDTYPE
88812	27318	T	T	A	I	L	S	G	F	L	L	H	V	Y	A	S	N	A	G	P	T	S	D	Q	S	Q	R	Y	D	S	P	A	S	V	
50179	0	T	I	D	T	L	–	–	–	L	L	–	–	–	A	S	Y	D	G	H	I	A	H	Q	S	*	I	C	L	S	P	A	F	V	
9670	620	T	T	A	I	L	S	G	F	L	L	H	V	Y	A	N	N	A	G	P	T	S	D	Q	S	Q	R	Y	D	S	P	A	S	V	
5370	848	T	T	A	I	F	S	G	F	L	L	H	V	Y	A	S	N	A	G	P	T	S	D	Q	S	Q	R	Y	D	S	P	A	S	V	
1174	0	T	I	D	T	L	–	–	–	L	F	–	–	–	A	S	Y	D	G	H	I	A	H	Q	S	*	I	C	L	S	P	A	F	V	
783	87	T	T	A	I	L	S	G	F	L	L	H	V	Y	A	S	N	A	G	H	T	S	D	Q	S	Q	R	Y	D	S	P	A	S	V	
720	4	T	T	A	I	L	S	G	F	L	L	–	–	Y	A	S	N	A	G	P	T	S	D	Q	S	Q	R	Y	D	L	P	A	S	V	
690	13	T	T	A	I	L	S	G	F	L	L	H	V	Y	A	S	N	A	G	P	T	S	D	Q	S	Q	R	Y	D	S	S	A	S	V	
636	1	T	T	A	I	L	S	G	F	L	L	H	V	Y	A	S	Y	A	G	P	T	S	D	Q	S	Q	R	Y	D	S	P	A	S	V	
401	12	T	T	A	I	L	S	G	F	L	L	H	V	Y	V	S	N	A	G	P	T	S	D	Q	S	Q	R	Y	D	S	P	A	S	V	
362	0	T	T	A	I	L	S	G	F	L	L	H	V	Y	A	R	N	A	G	P	T	S	D	Q	S	Q	R	Y	D	S	P	A	S	V	
342	0	T	I	D	T	F	–	–	–	L	L	–	–	–	A	S	Y	D	G	H	I	A	H	Q	S	*	I	C	L	S	P	A	F	V	
232	17	T	T	A	I	L	S	G	F	L	L	H	V	Y	A	S	N	A	G	P	T	S	D	Q	S	Q	R	Y	D	S	P	A	S	L	
223	99	T	T	A	I	L	S	G	F	L	F	H	V	Y	A	S	N	A	G	P	T	S	D	Q	S	Q	R	Y	D	S	P	A	S	V	
201	9723	T	T	A	I	L	S	G	F	L	L	H	V	Y	A	S	N	A	G	P	T	S	D	H	S	Q	R	Y	D	S	P	A	S	V	
165	11438	T	T	A	I	L	S	G	F	L	L	H	V	Y	A	S	N	A	G	P	T	S	D	Q	S	Q	R	Y	D	L	P	A	S	V	
156	82	T	T	A	I	L	S	G	F	L	L	H	V	–	A	S	N	A	G	P	T	S	D	Q	S	Q	R	Y	D	S	P	A	S	V	
148	5	T	I	A	I	L	S	G	F	L	L	H	V	Y	A	S	N	A	G	P	T	S	D	Q	S	Q	R	Y	D	S	P	A	S	V	
129	33	T	T	A	I	L	S	G	F	L	L	H	V	Y	A	S	N	A	G	P	T	S	D	Q	S	*	R	Y	D	S	P	A	S	V	
121	0	T	T	A	I	L	S	G	F	L	L	H	V	Y	A	I	N	A	G	P	T	S	D	Q	S	Q	R	Y	D	S	P	A	S	V	
114	5	T	T	A	I	L	S	G	F	L	L	H	V	Y	A	S	N	A	G	R	T	S	D	Q	S	Q	R	Y	D	S	P	A	S	V	
113	0	T	T	A	I	F	S	G	F	L	L	–	–	Y	A	S	N	A	G	P	T	S	D	Q	S	Q	R	Y	D	L	P	A	S	V	
113	1	T	T	A	I	L	F	G	F	L	L	H	V	Y	A	S	N	A	G	P	T	S	D	Q	S	Q	R	Y	D	S	P	A	S	V	
112	0	T	I	D	T	L	S	G	L	L	L	–	–	–	A	S	Y	D	G	H	I	A	H	Q	S	*	I	C	L	S	P	A	F	V	
103	237	T	T	A	I	L	S	G	F	P	L	H	V	Y	A	S	N	A	G	P	T	S	D	Q	S	Q	R	Y	D	S	P	A	S	V	
92	1	T	T	A	I	F	S	G	F	L	L	H	V	Y	A	N	N	A	G	P	T	S	D	Q	S	Q	R	Y	D	S	P	A	S	V	
88	12	T	T	A	I	L	S	G	F	L	L	H	V	Y	A	S	N	A	G	P	T	S	D	Q	S	Q	R	Y	Y	S	P	A	S	V	
87	106	I	T	A	I	L	S	G	F	L	L	H	V	Y	A	S	N	A	G	P	T	S	D	Q	S	Q	R	Y	D	S	P	A	S	V	
85	0	T	T	A	I	L	–	–	–	L	F	H	V	Y	A	S	Y	A	G	P	T	S	D	Q	S	Q	R	Y	D	S	P	A	S	V	
85	65	T	T	A	I	L	S	G	F	L	L	H	V	Y	A	S	N	A	D	P	T	S	D	Q	S	Q	R	Y	D	S	P	A	S	V	
81	17	T	T	A	I	L	S	G	F	L	L	H	V	Y	A	S	N	A	G	P	I	S	D	Q	S	Q	R	Y	D	S	P	A	S	V	
78	6	T	T	A	I	L	S	G	F	L	L	H	V	Y	A	S	N	A	G	L	T	S	D	Q	S	Q	R	Y	D	S	P	A	S	V	
58	6750	T	T	A	I	L	S	G	F	L	L	–	–	Y	A	S	N	A	G	P	T	S	D	Q	S	Q	R	Y	D	S	P	A	S	V	
57	1	T	T	A	I	L	S	G	F	L	L	H	F	Y	A	S	N	A	G	P	T	S	D	Q	S	Q	R	Y	D	S	P	A	S	V	
54	0	T	T	A	I	L	S	G	F	L	L	H	V	Y	A	S	N	A	G	P	T	S	Y	Q	S	Q	R	Y	D	S	P	A	S	V	
34	32622	I	T	A	I	L	S	G	F	L	L	H	V	Y	A	S	N	A	G	P	T	S	D	H	S	Q	R	Y	D	S	P	A	S	V	
17	11834	T	T	A	I	L	S	G	F	P	L	H	V	Y	A	S	N	A	D	P	T	S	D	Q	S	Q	R	Y	D	S	P	A	S	V	
15	5434	T	T	A	I	L	S	G	F	L	L	H	V	Y	A	S	N	A	G	P	T	S	D	Q	S	Q	R	Y	D	S	L	A	S	V	
KP 208		T	I	D	T	L	-	-	-	L	L	-	-	-	A	S	Y	D	G	H	I	A	H	Q	S	*	I	C	L	S	P	A	F	V	

**Highlighted are the defining variations of the B.1.1.7 variant. Note the K203/P204 variant shown at the bottom is likely to have arisen from the B.1.1.7 UK variant.*

Table S5. Variants R203/G204 and K203/R204 are the main amino acid combinations at positions 203 and 204 in nucleocapsid*.

203/204 amino acid	Count [codon combination]		% deposited sequences
RG	302305 [AGG] [GGA]	31 [AGA] [GGA]	62.2
KR	181752 [AAA] [CGA]		37.4
KL	915 [AAA] [CTA]		0.19
KG	414 [AAG] [GGA]		0.1
KP	240 [AAA] [CCA]		<0.1
MG	207 [ATG] [GGA]		<0.1
RR	98 [AGG] [AGA]		<0.1
SG	93 [AGT] [GGA]		<0.1

**Global SARS-CoV-2 sequences with sequence coverage of nucleocapsid amino acid positions 203 and 204 downloaded from www.gisaid.org on 24th of January 2021.*

Table S6. Frequency of sgRNA transcripts in 90 individuals that carry either the K203/R204 or R203/G204 variant from the SRA database (www.ncbi.nlm.nih/sra).*

45 KR Samples (leader sequence counts)			Sample Count 2 MMs	
Leader Start Base Position	TRS Start Base Position	Gene / Protein	Sample Count	Read Count
39	66	ORF1a	45	10087
3784	3811		1	1
5675	5702		1	2
10609	10636		1	1
14340	14367		1	1
18910	18937		1	1
21525	21552	S	17	125
22116	22143		1	1
23822	23849		2	2
25354	25381	ORF3a	9	13
26202-26208	26229-26235	E	19	93
26442	26469	M	13	44
27010	27037	ORF6	21	37
27356-27359	27383-27386	ORF7a	14	22
27643	27670		1	1
27857	27884	ORF8	1	2
28134	28161		1	1
28228-28233	28255-28260	N	40	704
28851	28878	N KR	5	6

TRS sequence counts		
ORF1a	45	57989
S	45	49243
ORF3a	40	150
E	33	380
M	45	114549
ORF6	31	187
ORF7a	39	306
ORF8	45	221224
N	45	288450
N KR	27	61

45 RG Samples (leader sequence counts)			Sample Count 2 MMs	
Leader Start Base Position	TRS Start Base Position	Gene / Protein	Per Sample	Read Count
39	66	ORF1a	45	14261
462	489		1	1
3784	3811		1	1
3886	3913		1	1
5561	5588		1	2
9562	9589		1	1
12938	12965		1	2
19081	19108		1	1
21040	21067		1	1
21525	21552	S	28	81
22470	22497		2	2
22528	22555		1	2
25354	25381	ORF3a	6	10
25391	25418		1	1
25633	25660		1	6
26206	26233	E	24	53
26260	26287		1	1
26442	26469	M	22	64
27010	27037	ORF6	22	44
27357	27384	ORF7a	6	10
27857	27884	ORF8	2	3
28228-28233	28255-28260	N	41	342
29350	29377		1	2

TRS sequence counts		
ORF1a	45	85214
S	45	44171
ORF3a	30	101
E	37	311
M	45	130459
ORF6	39	288
ORF7a	38	206
ORF8	45	239659
N	45	326435
N RG	0	0

**The top two tables represent matches spanning position 6 to 27 of the leader sequence with up to two mismatches. The bottom two tables represent a relaxation of the criterion for partial leader sequence matches to allow for the known poor quality sequence at the 5' end of sequence reads. Highlighted in red is the novel non-canonical nucleocapsid sgRNA.*

Table S7. Risk of admission to critical care unit according to age, sex and R203/G204 vs K203/R204 status of infecting SARS-CoV-2 strain.*

	Odds Ratio	95% CI	P value
Age in years	1.00	0.99 - 1.02	0.898
Sex (Male)	4.16	2.09 - 8.88	9.43E-05
K203/R204	1.20	0.63 - 2.34	0.588

**Multivariable logistic regression model using data from 981 individuals sampled in Sheffield, UK.*

Table S8. Impact of extraction method, day of illness at sampling and spike 614/nucleocapsid 203/204 variant on E gene cycle threshold (CT) value (A) G_RG and G_KR estimates using D_RG as reference and (B) D_RG and G_KR estimates using G_RG as reference.*

A

		**Estimate**	**95% CI**	**P value**
Extraction_method (heat inactivation)		3.76	2.94 – 4.58	<2.00E-16
Days from symptom onset		0.42	0.23 – 0.61	2.05E-05
Spike 614 & Nucleocapsid 203/204 status: **Reference D_RG (wild type)**	G_RG	-2.01	-3.12 to -0.68	0.00011
	G_KR	-1.90	-3.01 to -1.00	0.0023

B

		**Estimate**	**95% CI**	**P value**
Extraction_method (heat inactivation)		3.76	2.94 – 4.58	<2.00E-16
Days from symptom onset		0.42	0.23 – 0.61	2.05E-05
Spike 614 & Nucleocapsid 203/204 status: **Reference G_RG**	D_RG	1.90	0.68 – 3.12	0.0023
	G_KR	-0.16	-1.07 to 0.86	0.83

**Results from multivariable linear regression models. n=478 individuals sampled in Sheffield, UK). D_RG = D614/R203/G204; G_RG = G614/R203/G204; G_KR = G614/K203/R204. As due to reagent availability, method of extraction from clinical diagnostic samples changed during the study from the Magnapure96-based extraction to heat inactivation alone, this variable was included in the models. Heat inactivation (compared to Magnapure96 extraction) and later day from symptom onset were both associated with higher CT values (lower viral loads). K203/204 status is not associated with a change in CT value (A), whereas D614G status is associated with lower CT values/high viral loads (B). Of note K203/R204 samples form a 'subset' of D614G-containing variants.*

Table S9. Impact day of illness at sampling and spike 614/nucleocapsid 203/204 variant on total canonical sub-genomic RNA levels (A) G_RG and G_KR estimates using D_RG as reference and (B) D_RG and G_KR estimates using G_RG as reference.*

A

**sgRNA expression**		**Estimate**	**95% CI**	**P value**
Days from symptom onset		0.61	0.39 – 0.84	9.9E-08
Spike 614 & Nucleocapsid 203/204 status: **Reference D_RG (wild type)**	G_RG	-1.81	-3.20 to -0.41	0.011
	G_KR	0.58	-0.57 to 1.72	0.32

B

		**Estimate**	**95% CI**	**P value**
Days from symptom onset		0.61	0.39 – 0.84	9.9E-08
Spike 614 & Nucleocapsid 203/204 status: **Reference G_RG**	D_RG	1.81	0.41 – 3.20	0.011
	G_KR	2.38	1.24 – 3.52	4.51E-05

**Results from multivariable linear regression models. n=478 individuals sampled in Sheffield, UK. D_RG = D614/R203/G204; G_RG = G614/R203/G204; G_KR = G614/K203/R204.*
